# General practitioner care in nursing homes during the first wave of the COVID-19 pandemic in Germany: a retrospective survey among nursing home managers

**DOI:** 10.1186/s12875-022-01947-w

**Published:** 2022-12-22

**Authors:** Anja Kühl, Christian Hering, Wolfram J. Herrmann, Annabell Gangnus, Raphael Kohl, Elisabeth Steinhagen-Thiessen, Adelheid Kuhlmey, Paul Gellert

**Affiliations:** 1 Institute of Medical Sociology and Rehabilitation Science, Charité - Universitätsmedizin Berlin, Berlin, Germany; 2 Institute of General Practice and Family Medicine, Charité - Universitätsmedizin Berlin, Berlin, Germany; 3 Department of Endocrinology, Diabetes and Metabolism, Charité - Universitätsmedizin Berlin, Berlin, Germany

**Keywords:** General practitioners, Primary care physicians, Nursing homes, COVID-19, Visiting restrictions, Healthcare provision, Primary care

## Abstract

**Background:**

Though evidence on the detrimental impact of the COVID-19 pandemic in nursing homes is vast, research focusing on general practitioners’ (GP) care during the pandemic in nursing homes is still scarce.

**Methods:**

A retrospective online survey among 1,010 nursing home managers in Germany was conducted during the first wave of the COVID-19 pandemic between November 2020 and February 2021. Associations between perceived deficits in GP care (routine and acute visits) and both general and COVID-19-related characteristics of nursing homes were analysed using multiple logistic regression analyses.

**Results:**

The majority of nursing home managers reported no deficits in GP care (routine visits, 84.3%; acute visits, 92.9%). Logistic regression analyses revealed that deficits in GP care (routine visits) were significantly associated with visiting restrictions for GPs and nursing home size. Small nursing homes (1–50 residents) were significantly more likely to report deficits in GP care (routine visits) compared to medium (51–100 residents) and large nursing homes (> 100 residents). Further, deficits in GP care (acute visits) were significantly associated with dementia as a focus of care and the burden of insufficient testing for SARS-CoV-2 among residents. Moreover, visiting restrictions for GPs were significantly associated with dementia as the focus of care and the COVID-19 incidence at the federal state level. Finally, COVID-19 cases in nursing homes were significantly associated with size of nursing homes, COVID-19-incidence on the federal state level and the burden of insufficient testing capacities for SARS-CoV-2 among residents.

**Conclusion:**

We found structural factors associated with GP care deficits during the pandemic. New concepts for GP care should be implemented in pandemic preparedness plans to ensure high quality, consistent, and reliable GP care as well as effective infection prevention measures in nursing homes.

## Background

The novel coronavirus, initially called 2019-nCov [1], has been spreading around the world [2] since it was discovered in Wuhan, China, in December 2019 [1]. On February 11, 2020, the World Health Organisation (WHO) announced that this new coronavirus would be called “severe acute respiratory syndrome coronavirus 2” (SARS-CoV-2) and named the disease it causes ‘COVID-19’ [3]. The first COVID-19 case in Germany occurred at the end of January 2020 [4]; by March 2020, the WHO had declared the global expansion of SARS-CoV-2 a pandemic [2, 5]. Soon, it became clear that chronically ill and elderly people in long-term care facilities, such as nursing homes, are exceptionally vulnerable to COVID-19 due in part to the congregant living situation found in nursing homes [6, 7]. During the first wave of the COVID-19 pandemic, the Robert Koch Institute (RKI) announced that, in Germany, 86% of deaths occurred in persons aged 70 years or older [8]. International research shows that the mortality of hospitalised patients with COVID-19 above the age of 80 years is up to 54% [9]. Comas-Herrera et al. estimated that, on average, across 26 countries, approximately 47% of all COVID-19 deaths were among nursing home residents during the first three months of the pandemic [10].

During the pandemic, structural deficits in the long-term care system in Germany gained visibility. Staffing shortages, frequent staff turnover, low preparedness for infection control, and inadequate infection prevention were all observed by researchers [11–13]. General practitioners (GPs) are key providers for primary care and typically have frequent contact with nursing home residents [14–16]. However, GP provision of palliative care, wound management, and mental healthcare for nursing home residents in Germany was already reported as insufficient before the pandemic [15, 16].

As with structural deficits, the frequency of consultations between nursing home residents and GPs and medical specialists was inconsistent and less frequent for residents of long-term care facilities than for the general population of the same age even before the pandemic began [16–20]. For instance, Kleina et al. [18] reveal in their cross-sectional survey among 778 residents from 8 nursing homes high deficits in general and specialised healthcare of nursing home residents in Germany. In 2017 they collected data on the health status and healthcare in order to investigate the current situation regarding healthcare for nursing home residents in Germany. Even though Kleina et al. [18] were able to show that about 91% of the residents had personal contact to GPs or internists quarterly, the frequency of contacts to other medical specialists, especially urologists (18.9% of the residents) and ophthalmologists (16.7% of the residents) within a year was lower compared to people the same age not living in nursing homes. Further, Schröder et. al. [16] described in a cross-sectional survey among German nursing homes staff, conducted in 2019, a large variance in the number of GPs caring per nursing home resident. They also stated that nursing homes are in contact with many different GPs, which might prevent residents from more regular visits by a certain GP. However, Schröder et al. [16] point out that regular visits are supposed to be a key factor in improving the healthcare for nursing home residents. Conclusively, deficits in the general and specialised healthcare in nursing homes existed even before the pandemic. However, it is unclear how much of an impact the pandemic has had on this aspect of the healthcare system in Germany.

Infection prevention measures against the coronavirus, i.e. visiting restrictions at hospitals and nursing homes, were enacted in many countries [21, 22]. In Germany, nursing homes were urged to quickly adapt by implementing new hygiene guidelines for infection control and arranging visiting restrictions; nursing home staff experienced both of these procedures as burdensome [23]. For care providers such as GPs, visiting restrictions aggravated their work burden during the pandemic [21]. GPs were faced with the daunting task of communicating with family members, providing psychosocial support, as well as maintaining routine and acute healthcare for residents without elevating the risk of SARS-CoV-2 infections [21]. In advance, Hugelius et al. [21] indicate in an integrative review how visiting restrictions has caused healthcare providers, including GPs, to suffer from moral distress including guilt, powerlessness and insufficiency [21].

Therefore, the primary aim of the present study is to investigate whether nursing home managers perceived deficits in GP care during the first wave of the pandemic. Further, this study evaluates COVID-19-related and structural factors associated with perceived deficits in GP care.

## Methods

### Design and sampling

A retrospective online survey was conducted between November 15, 2020, and February 28, 2021, using the Research Electronic Data Capture System (REDCap), a secure web application. The targeted time frame of the survey was between March 1, 2020, and June 30, 2020, so as to collect information about the first wave of the pandemic and the following “lockdown” in Germany. The survey is part of the interdisciplinary COVID-Heim project, which aims to draw lessons from the pandemic for structural developments in the nursing home setting in Germany by combining various data sources.

In Germany, there is a total of 15,380 long term care facilities, including daytime inpatient nursing care and permanent inpatient nursing care for older people [24]. For the present study, a contact list of 11,317 nursing homes providing permanent inpatient nursing care was made available by the AOK research institute (WIdO). A total of 10,026 nursing homes were contacted via email, and a further 1,291 nursing homes without specified email addresses were contacted via mail. An invitation to participate in our survey containing a link and QR-Code was sent to nursing home managers. Altogether, the questionnaire was opened 1,973 times and completed by 886 nursing home managers (44.9%). Finally, 1,010 nursing home managers provided data about GP care and were included in our analysis. Therefore, the response rate was 8.9%. The research design, an anonymous online survey, was approved by the ethics committee of the Faculty of Medicine of the Charité – Universitätsmedizin Berlin (EA1/254/20).

### Measures

#### General characteristics of nursing homes

We asked nursing home managers to report on the general characteristics of homes they managed, including size (small, 1–50 residents; medium, 51–100 residents; or large, > 100 residents) and ownership (private, non-profit, or public). Furthermore, we inquired about the existence of a cooperation or contract with a GP (yes/no) and the presence of an employed GP in the facility (yes/no). In Germany the primary healthcare in nursing homes is almost exclusively provided by GPs [15, 16, 25, 26], whereas GPs, medical specialists and allied health professionals are not regularly employed by nursing homes [16]. Basically, three different organizational concepts of primary healthcare in nursing homes in Germany are established: 1) An employed GP in the facility, who is responsible for the entire primary healthcare of all residents who do not have their own registered GP in a local private practice; 2) Contract with a GP: The nursing home concludes a contract with a small number of GPs, in which certain services that the GPs should provide for the residents (e.g. routine visits, acute visits, consultation hours in the nursing home, palliative care) are specified; and 3) Cooperation with a GP: A local GP is contractually bound to the facility in order to improve the cooperation between the nursing home and GPs in the local area [26]. Finally, we asked nursing home managers whether dementia is the focus of care of their facility (yes/no). All items were adapted from Wolf-Ostermann et al. [27].

#### Covid-19-related characteristics

COVID-19 incidence on the state level. For local COVID-19 incidence in the general population, we used RKI daily situation reports for each state (*Bundesstaat*) in Germany (cases per 100,000 population in each state), beginning with the first report from March 4, 2020, [28] and ending with June 30, 2020, report, [29], the last day of our targeted time frame in the survey. We accumulated all confirmed COVID-19 cases up to June 30, 2020, as outlined above, to a cumulative incidence of cases per 100,000 population [29]. We categorized the COVID-19 incidence on the state level into four groups; 1–99; 100–199; 200–299; and > 300 cases per 100,000 population. The cumulative incidence (per 100,000 population) of confirmed COVID-19 cases for each state in Germany of the targeted time frame can be found in Table 1 [29].

**Table 1:** COVID-19 incidence in Germany (March 1, 2020 – June 30, 2020) [29]

State	Total number of cases	Cases / 100.000 population
Baden-Württemberg	35,600	321.8
Bavaria	48,400	370.1
Berlin	8,220	219.3
Brandenburg	3,429	136.5
Bremen	1,662	243.3
Hamburg	5,201	282.5
Hesse	10,795	172.3
Mecklenburg-Western Pomerania	802	49.8
Lower Saxony	13,535	169.6
North Rhine-Westphalia	43,066	240.2
Rhineland-Palatinate	6,996	171.3
Saarland	2,806	283.3
Saxony	5,448	13.6
Saxony-Anhalt	1,871	84.7
Schleswig–Holstein	3,154	108.9
Thuringia	3,254	151.8
**Total**	**194,259**	**234**

COVID-19-related burden. COVID-19 related burden experienced by nursing home managers were assessed on a four-point Likert scale: no burden (0), moderate burden (1), strong burden (2), and very strong burden (3). The items presented for evaluation were: 1) acquisition and utilisation of infection control equipment (e.g. masks, protective clothing); 2) compliance with RKI hygiene guidelines; 3) economic problems (e.g. staff shortage, deviations from the working time act); 4) insufficient testing for SARS-CoV-2 infections among residents; and 5) concerns about SARS-CoV-2 infections among residents. The single items were adapted from Hower et al. [23].

COVID-19 impact on GP care (outcome). COVID-19 cases among residents or staff were assessed by asking nursing home managers whether there were any COVID-19 cases among residents or staff since March 2020 (yes/no). Further, visiting restrictions for GPs were assessed by asking nursing home managers how access to facility was regulated for GPs during the 1^st^ of March, 2020 to 30^th^ of June 2020. Possible answers were that GPs had 1) unrestricted access; 2) access with limitations; meaning that GPs were only granted access in the event of an emergency 3) no access; or 4) not applicable; meaning that there is no external GP visiting residents [27]. Further, nursing home managers reported on deficits in GP care in their facilities for routine visits and acute visits respectively on a four-point Likert scale: strongly disagree (0), partially disagree (1), partially agree (2), and strongly agree (3). The two items used to assess deficits in GP care were designed by an interdisciplinary team consisting of psychologists, sociologists and medical doctors who are professionals regarding challenges of primary care in long-term care facilities. Moreover, this is also common practice in ad-hoc survey research [30]. We used a four-point Likert scale without a neutral option so nursing home managers were required to commit to an answer (i.e. forced choice). In order to perform logistic regression analysis all outcomes were dichotomized as explained in the section statistical analysis.

### Statistical analysis

Descriptive statistics were used to generate frequencies and percentages for general characteristics of nursing homes, COVID-19 related characteristics of nursing homes and COVID-19 impact on GP care. We used multiple logistic regression analysis to regress COVID-19 impact on GP care (outcome) on potentially associated factors, which were COVID-19 cases in nursing homes (yes vs. no), visiting restrictions for GPs (no access/access with limitations vs. unrestricted access), and deficits in GP care for routine visits (strongly/partially agree vs. strongly/partially disagree) and GP care for acute visits (strongly/partially agree vs. strongly/partially disagree). Missing values were excluded from the analysis. Statistical analyses were performed using IBM SPSS Statistics for Windows, version 27.0 (IBM Corp., Armonk, NY). Statistical significance was assumed at *p* < 0.05.

## Results

### General and COVID-19-related characteristics of nursing homes

The majority of participating nursing homes were either small (1–50 residents, *n* = 279, 27.6%) or medium-sized (51–100 residents, *n* = 500, 49.5%). Likewise, about half of participating nursing homes were in non-profit ownership (50.4%, *n* = 509) and four in ten nursing homes were in private ownership (40.5%, *n* = 409). Further, 866 out of 1,010 (85.7%) participating nursing home managers reported having cooperation contracts with GPs. An additional 2% (*n* = 20) managers reported employing in-house GPs. Furthermore, 119 (11.8%) of nursing homes reported dementia as their focus of care. The greatest COVID-19-related burden was ‘concerns about SARS-CoV-2 infections among residents’ (strong/very strong burden, *n* = 896; 88.7%), followed by ‘compliance with hygiene guidelines of Robert Koch- Institute’ (strong/very strong burden, *n* = 536; 53.1%). Approximately two-fifths of the nursing homes perceived ‘economic problems ‘ (*n* = 443, 43.9%) and ‘insufficient testing for SARS-CoV-2 infections among residents’ (*n* = 438, 43.4%) as strong or very strong burdens. General and COVID-19-related characteristics of nursing homes are shown in detail in Table 2.

**Table 2:** General and COVID-19-related characteristics of nursing homes (*N* = 1,010)

Variable	**n**	**%**
**General characteristics of nursing homes**		
Size		
Small (1–50 residents)	279	27.6
Medium (51–100 residents)	500	49.5
Large (> 101 residents)	219	21.7
Not specified/missing	12	1.2
Ownership		
Private	409	40.5
Non-Profit	509	50.4
Public	85	8.4
Not specified/missing	7	0.7
Cooperation/Contract with GP		
Yes	866	85.7
No	144	14.3
Not specified/missing	0	0
Employed GP in a facility		
Yes	20	2.0
No	980	97.0
Not specified/missing	10	1.0
Dementia as a focus of care		
Yes	119	11.8
No	891	88.2
Not specified/missing	0	0
**Covid-19-related characteristics**		
COVID-19 Incidence on the state level in Germany^a^		
1–99	64	6.3
100–199	375	37.1
200–299	297	29.4
> 300	274	27.1
COVID-19-related burden		
*Acquisitions and utilisation of infection control equipment (masks, protective clothing, *etc*.)*		
None/moderate burden	602	59.6
Strong/very strong burden	357	35.3
Not specified/missing	51	5.0
*Compliance with hygiene guidelines of RKI*		
None/moderate burden	453	44.9
Strong/very strong burden	536	53.1
Not specified/Missing	21	2.1
*Economic Problems (staff shortage, deviations from the working time act*)		
None/moderate burden	545	54.0
Strong/very strong burden	443	43.9
Not specified/missing	22	2.2
*Insufficient testing for SARS-CoV-2 infections among residents*		
None/moderate burden	541	53.6
Strong/very strong burden	438	43.4
Not specified/missing	31	3.1
*Concerns about SARS-CoV-2 infections among residents*		
None/moderate burden	90	8.9
Strong/very strong burden	896	88.7
Not specified/missing	24	2.4
**COVID-19 impact on GP care (outcome)**		
COVID-19 cases in nursing homes		
Yes	261	25.8
No	730	72.3
Not specified/missing	19	1.9
Visiting restrictions for GPs		
Unrestricted access	659	65.2
No access/access with limitations	341	33.8
Not specified/missing	10	1.0
Deficits in GP care for routine visits		
Strongly/partially agree	159	15.7
Strongly/partially disagree	851	84.3
Not specified/missing	0	0
Deficits in GP care for acute visits		
Strongly/partially agree	66	6.5
Strongly/partially disagree	938	92.9
Not specified/missing	6	0.6

*GP* General practitioner, *RKI* Robert Koch institute

^a^ Cases/100.000 pop., March 1 – June 30, 2020

^b^ COVID-19 cases among residents or staff

### The impact of COVID-19 on GP care (outcome)

In 25.8% (*n* = 261) of all nursing homes COVID-19 cases among residents or staff occurred. In about two-thirds (65.2%, *n* = 659) of nursing homes, GPs had unrestricted access to the facility. In the remaining third (33.8%) of participating nursing homes, access for GPs was limited or not possible at all. Nursing home managers reported more deficits in GP care for routine visits (15.7%, *n* = 159) than for the provision of GP care for acute visits (6.5%, *n* = 66). Most nursing homes reported no deficits in GP care for routine visits (84.3%) or acute visits (92.9%, see Table 2).

### Multivariate results

Multiple logistic regression analysis were performed to identify factors associated with COVID-19 cases in nursing homes (yes vs. no), visiting restrictions for GPs (no access/access with limitations vs. unrestricted access), and deficits in GP care for routine visits (strongly/partially agree vs. strongly/partially disagree) and GP care for acute visits (strongly/partially agree vs. strongly/partially disagree). Results showed that COVID-19 cases in nursing homes (yes) were linked to the size of nursing homes, whereas medium and large nursing homes were more likely to report COVID-19 cases in their facility compared to small nursing homes (OR 1.9, CI 1.2 – 2.8, *p* < 0.01; OR 4.0, CI 2.5 – 6.3, *p* < 0.001). In addition, COVID-19 cases in nursing homes were associated with the COVID-19 incidence on the state level in Germany. Facilities located in states with a COVID-19 incidence rate of 200 or higher were more likely to report COVID-19 cases in their facility compared to facilities in states with an incidence of 1–99 (OR 3.2, CI 1.3 – 7.9, *p* =0.013; OR 3.9, CI 1.6 – 9.8, *p* =0.004). Finally, COVID-19 cases in nursing homes were associated with a strong/very strong burden due to insufficient testing for SARS-CoV-2 infection among residents (OR 1.6, CI 1.1 – 2.1, *p* < 0.01).

Moreover, logistic regression analysis revealed that deficits in GP care for routine visits (strongly/partially agree) – but not acute visits – were significantly linked to the size of nursing homes. Large-sized nursing homes (OR 0.6, CI 0.3 – 1.0; *p* = 0.042) experienced significantly less deficits in GP care for routine visits (strongly/partially agree) than in smaller nursing homes. Furthermore, deficits in GP care for routine visits were linked to visiting restrictions for GPs (no access/access with limitations; OR 2.1, CI 1.4 – 3.1, *p* < 0.001). Moreover, deficits in GP care for acute visits (strongly/partially agree) was associated with dementia as the focus of care (OR 2.1, CI 1.0 – 4.4; *p* = 0.039) and a strong/very strong burden due to insufficient testing for SARS-CoV-2 infections among residents (OR 1.9, CI 1.1 – 3.5; *p* = 0.029).

Lastly, visiting restrictions for GPs (no access/access with limitations) were associated with a COVID-19 incidence of at least 300 on the state level (OR 2.3, CI 1.2 – 4.5, *p* =0.011) and dementia as focus care (OR 1.6, CI 1.0 – 2.5, *p* =0.029). Results from the multiple logistic regression analysis are shown in Table 3.

**Table 3:** Relationship between general and COVID-19-related characteristics of nursing homes and COVID-19 cases, visiting restrictions for GPs, and deficits in GP care

Variable	**COVID-19 cases in nursing homes (yes)**^**1**^		**Visiting restrictions for GPs (no access/access with limitations)**^**2**^		**Deficits in GP care for routine visits (strongly/partially agree)**^**3**^		**Deficits in GP care for acute visits (strongly/partially agree)**^**4**^	
	OR (95% CI)	*p*	OR (95% CI)	*p*	OR (95% CI)	*p*	OR (95% CI)	*p*
**General characteristics of nursing homes**								
Size								
Small (1–50 residents)	-	-	-	-	-	-	-	-
Medium (51–100 residents)	**1.9 (1.2 – 2.8)**	**0.003**	0.9 (0.6 – 1.2)	0.484	0.7 (0.4 – 1.0)	0.050	0.9 (0.5 – 1.7)	0.739
Large (> 100 residents)	**4.0 (2.5 – 6.3)**	** < 0.001**	0.8 (0.5 – 1.2)	0.332	**0.6 (0.3 – 1.0)**	**0.042**	0.5 (0.2 – 1.2)	0.100
Ownership								
Private	-	-	-	-	-	-	-	-
Non-Profit	0.9 (0.5 – 1.7)	0.838	1.3 (0.8 – 2.2)	0.332	1.3 (0.7 – 2.5)	0.453	1.5 (0.6 – 3.7)	0.381
Public	0.9 (0.7 – 1.3)	0.675	0.9 (0.7 – 1.3)	0.698	1.1 (0.7 – 1.6)	0.808	0.7 (0.4 – 1.3)	0.309
Cooperation/Contract with GP (yes)	0.7 (0.5 – 1.1)	0.107	0.8 (0.5 – 1.1)	0.186	0.9 (0.6 – 1.5)	0.750	0.5 (0.3 – 1.0)	0.066
Employed GP in facility (yes)	0.7 (0.2 – 2.4)	0.603	0.7 (0.2 – 2.0)	0.479	1.2 (0.3 – 4.4)	0.776	1.2 (0.1 – 10.0)	0.874
Dementia as focus of care (yes)	1.0 (0.6 – 1.7)	0.880	**1.6 (1.0 – 2.5)**	**0.029**	1.6 (1.0 – 2.7)	0.073	**2.1 (1.0 – 4.4)**	**0.039**
**COVID-19-related characteristics**								
COVID-19 incidence on the state level in Germany^a^								
1–99	-	-	-	-	-	-	-	-
100–199	2.4 (1.0 – 5.9)	0.062	1.1 (0.6 – 2.0)	0.863	1.6 (0.7 – 4.1)	0.289	0.9 (0.3 – 2.8)	0.832
200–299	**3.2 (1.3 – 7.9)**	**0.013**	0.9 (0.5 – 1.8)	0.850	1.1 (0.4 – 2.8)	0.890	0.6 (0.2 – 2.0)	0.391
> 300	**3.9 (1.6 – 9.8)**	**0.004**	**2.3 (1.2 – 4.5)**	**0.011**	1.3 (0.5 – 3.4)	0.539	0.5 (0.2 – 1.8)	0.308
COVID-19 cases in nursing homes (yes)^b^	-	-	0.9 (0.6 – 1.2)	0.511	1.5 (1.0 – 2.3)	0.058	1.7 (0.9 – 3.0)	0.100
COVID-19-related burden								
*Acquisition and utilisation of infection control equipment (masks, protective clothing, *etc*.)*								
None/moderate burden	-	-	-	-	-	-	-	-
Strong/very strong burden	0.7 (0.5 – 1.0)	0.068	1.1 (0.8 – 1.5)	0.539	1.3 (0.9 – 2.0)	0.139	1.5 (0.8 – 2.6)	0.188
*Compliance with hygiene guidelines of RKI*								
None/moderate burden	-	-	-	-	-	-	-	-
Strong/very strong burden	1.2 (0.8 – 1.6)	0.379	1.0 (0.7 – 1.3)	0.978	1.1 (0.8 – 1.6)	0.618	1.6 (0.9 – 3.0)	0.127
*Economic Problems (staff shortage, deviations from the working time act)*								
None/moderate burden	-	-	-	-	-	-	-	-
Strong/very strong burden	1.3 (1.0 – 1.8)	0.085	1.0 (0.8 – 1.4)	0.921	1.4 (0.9 – 2.0)	0.104	1.7 (0.9 – 3.0)	0.090
*Insufficient testing for SARS-CoV-2 infections among residents*								
None/moderate burden	-	-	-	-	-	-	-	-
Strong/very strong burden	**1.6 (1.1 – 2.1)**	**0.007**	1.1 (0.8 – 1.4)	0.680	1.0 (0.7 – 1.5)	0.946	**1.9 (1.1 – 3.5)**	**0.029**
*Concerns about SARS-CoV-2 infections among residents*								
None/moderate burden	-	-	-	-	-	-	-	-
Strong/very strong burden	1.4 (0.8 – 2.6)	0.274	1.0 (0.6 – 1.7)	0.965	1.5 (0.7 – 3.3)	0.307	1.9 (0.5 – 8.4)	0.375
Visiting restrictions for GPs (yes)	0.9 (0.6 – 1.2)	0.480	-	-	**2.1 (1.4 – 3.1)**	** < 0.001**	1.0 (0.6 – 1.8)	0.974

*OR* Odds ratio, *CI* Confidence interval, *GP* General practitioner, *RKI* Robert Koch institute

^a^ Cases/100,000 pop., March 1 – June 30, 2020

^b^ COVID-19 cases among residents or staff

^1^ yes vs. no

^2^ no access/access with limitations vs. unrestricted access

^3^ strongly/partially agree vs. strongly/partially disagree

^4^ strongly/partially agree vs. strongly/partially disagree; Significant values are shown in bold type

## Discussion

The present study investigates perceived deficits in GP care and associated factors during the first wave of the COVID-19 pandemic in German nursing homes. We found that the majority of nursing home managers reported no deficits in GP care (routine visits, 84.3%; acute visits, 92.9%). Still, deficits in GP care (routine visits) were associated with visiting restrictions for GPs and the size of the nursing homes. Small nursing homes (1–50 residents) were more likely to report deficits in GP care (routine visits) compared to medium (51–100 residents) or large nursing homes (> 100 residents). Further, deficits in GP care (acute visits) were associated with dementia as the focus of care and the burden of insufficient testing for SARS-CoV-2 among residents. Visiting restrictions for GPs were associated with dementia as the focus of care and COVID-19 incidence at the state level. Finally, COVID-19 cases in nursing homes were associated with the size of the facility, COVID-19-incidence at the state level, and the burden of insufficient testing for SARS-CoV-2 among residents.

Prior evidence indicated major challenges for nursing homes and in GP healthcare due to the pandemic [21, 31–34]. In the scoping review by Giri et al. [35], which includes 76 articles that were published between 1 March 2020 and 31 January 2021, multiple factors have been identified that simultaneously contributed to the individual challenges for nursing homes during the pandemic. These challenges include characteristics of the disease COVID-19 (e.g. asymptomatic transmission), resident related factors (e.g. comorbidities), structural characteristics of the facilities (e.g. size), staffing (e.g. staffing level) and external factors (e.g. availability of personal protective equipment) [35]. Further research findings describe negative effects of COVID-19-related burden, such as insufficient testing for SARS-CoV-2 infections [23, 31] and visiting restrictions [31], in the work environment of nursing and the ensuring of healthcare for residents. Considering all these evidence, the majority of nursing home managers in our sample did not report deficits in GP care during the first wave of the pandemic in Germany. Nevertheless, primary care in nursing homes had been a noticeable problem even before the pandemic began [15, 16], and even a slight worsening may have had a greater impact on residents as perceived by nursing home managers. Further, deficits in GP care may have been unevenly distributed over time, i.e., lacking during certain weeks of the pandemic and then compensated in the subsequent weeks.

In line with similar results from previous studies [31, 35], our findings imply that insufficient testing for SARS-CoV-2 infections among residents seems to be associated with COVID-19 cases and more deficits in GP care for acute medical cases in nursing homes. Prior research demonstrated that SARS-CoV-2 positive nursing home residents with asymptomatic cases can still contribute to the transmission of the coronavirus in long-term care facilities [10, 31, 36]. This research indicates that regular testing of residents and staff, regardless of the occurrence of symptoms, helps determine the true impact of COVID-19 [10] and is both desirable and recommended [31]. However, insufficient testing capacities appeared to be a major problem for infection prevention during the first wave of the COVID-19 pandemic in Germany, ultimately leading to a high burden for nursing home staff [23, 31].

In contrast to previous findings from Rothgang et al. [31], our results suggest that COVID-19 cases are more likely to be found in medium and large nursing homes (> 50 residents) in Germany. However, our results support research from the USA [37], Canada [38], and Spain [39, 40]. For example, in a study of 9,395 nursing homes in the USA, Abrams et al. [37] showed that larger facility size, urban location, and state were significantly related to an increased probability of having COVID-19 cases in nursing homes. In a cross-sectional analysis of nursing homes in Spain between March 1 and June 30, 2020, Soldevila et al. [40] found that larger nursing homes had a greater likelihood of a COVID-19 outbreak compared to their smaller counterparts (88.1% versus 37.0%, *P* < 0.001). Soldevila and colleagues [40] argued that large nursing homes were more vulnerable to a SARS-CoV-2 transmission due to the higher number of visiting relatives and working staff [40]. Furthermore, we found that a large facility size was linked to fewer deficits in GP care for routine visits. Though our results indicate that smaller nursing homes were less likely to have COVID-19 outbreaks, nursing home managers perceived more deficits in GP care (for routine visits but not acute visits) in these settings compared to medium and large nursing homes. One explanation could be that larger nursing homes might have a greater ability to provide a sufficient amount of nursing staff able to look after their residents and intervene at an early stage so that GPs don’t need to be consulted and no deficits in GP care occur. Otherwise, this unanticipated result may be attributed to the fact that GPs are probably able to see more patients at once during their routine visits to larger nursing homes. This simplicity of spatial opportunity and time-saving for GPs may emerge as a probable explanation for the lack of deficits in GP care for routine visits in larger care settings.

In our sample, almost one-third of nursing homes implemented visiting restrictions for GPs. This is comparable with previous findings by Rothgang and colleagues [31], who described among their surveyed German nursing homes that approximately one quarter did not allow access for external service providers (including GPs), and two-thirds only allowed access with limitations. Likewise, our results show that visiting restrictions for GPs were associated with perceived deficits in GP care for routine visits. In an exploratory Dutch study [41], most physicians providing care for residents in nursing homes described visiting restrictions as an ethical dilemma wherein they balanced safety as mediated through infection prevention measures and liveability for the residents, i.e. compensating for the absence of face-to-face contact [41].

Further, our data imply that nursing homes with dementia as a focus of care were particularly burdened by the impacts of the pandemic, which is in line with prior research conducted by Gordon et al. [42], who highlighted the COVID-19 related challenges of isolation and visiting restrictions especially for residents with cognitive impairments. Gordon et al. [42] further stated, that infection prevention measures resulted in further loss of autonomy and social isolation and especially residents with cognitive impairment were at risk of falls and injury due to a lack of supervision [42].

Furthermore, the association found between local COVID-19 incidence and COVID-19 cases in nursing homes in our data is also reflected in recent research out of the USA [37], Canada [38], and Spain [40]. In this context Soldevila et al. [40] argue that a high incidence in the general population raises the possibility of virus transmission into nursing homes by nursing home staff and visiting relatives [40]. Further, deficits in GP care (acute visits) were especially reported in nursing homes that experienced insufficient testing for SARS-CoV-2 infections among residents as a strong or very strong burden. This was also the case in nursing homes with dementia as the primary focus of care. Similarly, Grimm et al. [43] showed in their retrospective, cross-sectional analysis using linked administrative data that nursing home residents’ hospital admissions – including emergency admissions for acute coronary syndromes and stroke – declined during the first wave of the COVID-19 pandemic in England, potentially resulting in substantial unmet health issues [43].

### Strengths and limitations

Our study has both strengths and limitations. Strengths include the population-based design of the sampling. To our knowledge, the sample of the present study is the largest and most comprehensive sample of German nursing homes used to investigate the first wave of the COVID-19 pandemic; moreover, compared to all nursing homes in Germany our sample was comparable distributed in terms of ownership, but medium and large nursing homes were overrepresented in our sample [24]. Furthermore, taken the response rate of 8.9% into account generalizability may be limited, if response is considered selective. Nevertheless, the response rate is comparable to previous research focusing on the impact of COVID-19 on long-term care facilities in Germany [27]. Finally, time is an essential resource in nursing—especially during the pandemic, also explaining a lack of participation or drop out. Thus, a high dropout rate is a typical issue of online surveys [44]. Further, because of the nature of a retrospective survey, a potential recall bias needs to be taken into account. Since the second wave of the pandemic had a more detrimental impact on German nursing homes than the first, it is possible that the first wave of the pandemic was remembered as easier to manage, or even the opposite. Nursing homes may have adapted between the first and second waves regarding the lack of personal protection equipment, testing devices, and the overwhelming experience of the pandemic. It is thus possible that deficits in GP care may be perceived less strongly by the surveyed nursing home managers given the challenging context. Second, a selection bias toward nursing homes that are less affected by the pandemic should be considered.

Moreover, the present study only investigates perceived deficits in GP care by managers in nursing homes; this does not cover inadequacies in the utilisation of other aspects of the healthcare system, such as medical specialists [16–20], which was beyond the focus of the present study. Furthermore, we measured neither the quantity nor the quality of GP care during the pandemic, and the results may be prone to biases. Nevertheless, data does not indicate that probable differences are due to unreliable answers from nursing home managers. Future studies should include validated questionnaires to evaluate GP care during the pandemic more precisely.

Finally, even though we took the cumulative incidence of COVID-19 cases from the state level into account, we were not able to differentiate between urban and rural areas, which could provide valuable context in the light of previous research [37] that found a significant relationship between the urban location of nursing homes and an increased rate of new COVID-19 infections.

## Conclusion

The results of the present study provide new and valuable information on GP care in nursing homes during the first wave of the COVID-19 pandemic that helps to illuminate the diverse impacts of this extended health crisis. In particular, our data indicate that perceived deficits in GP care for routine and acute visits are more frequent in nursing homes with dementia as a focus of care; these homes should be supported by policymakers and legislation in the context of infection control, staffing and structural expansion and strategies to improve care should be enhanced as such. For example, new concepts of interprofessional collaboration between all care providers for nursing home residents could reduce negative outcomes. The development of nationwide recommendations for nursing homes during periods of elevated risk — like a pandemic — could provide reassurance and represents an important task for the RKI, the Federal Ministry of Health, and nursing care insurance companies. More broadly, structural factors were related to care deficits and therefore need to be considered when establishing pandemic action plans in the future. Mandatory training in geriatric medicine and gerontopsychiatry for GPs, consistent availability of GPs and medical specialists, and telemedicine techniques should be promoted so as to maintain high-quality primary care even during protection measurements like visiting restrictions. Likewise, structured guidelines for behaviour and hygiene standards need to be established and utilised for infection control during periods of increased disease transmission and beyond. Further, personal protection equipment and testing devices are needed to contribute to the improvement of GP care for this vulnerable group of patients who are especially in need of reliable and compassionate care.

## Data Availability

The datasets generated and/or analysed during the current retrospective study are available from the corresponding and senior authors on reasonable request.
